# Cells Grouping Detection and Confusing Labels Correction on Cervical Pathology Images

**DOI:** 10.3390/bioengineering12010023

**Published:** 2024-12-30

**Authors:** Wenbo Pang, Yi Ma, Huiyan Jiang, Qiming Yu

**Affiliations:** 1 Software College, Northeastern University, Shenyang 110819, China; 2010498@stu.neu.edu.cn (W.P.); 2410602@stu.neu.edu.cn (Q.Y.); 2 Information and Engineering College, Wenzhou Medical University, Wenzhou 325035, China; 3 Key Laboratory of Intelligent Computing in Medical Image, Ministry of Education, Northeastern University, Shenyang 110819, China

**Keywords:** data augmentation, grouping detection, noise sample, cervical cytology, pathological image

## Abstract

Cervical cancer is one of the most prevalent cancers among women, posing a significant threat to their health. Early screening can detect cervical precancerous lesions in a timely manner, thereby enabling the prevention or treatment of the disease. The use of pathological image analysis technology to automatically interpret cells in pathological slices is a hot topic in digital medicine research, as it can reduce the substantial effort required from pathologists to identify cells and can improve diagnostic efficiency and accuracy. Therefore, we propose a cervical cell detection network based on collecting prior knowledge and correcting confusing labels, called PGCC-Net. Specifically, we utilize clinical prior knowledge to break down the detection task into multiple sub-tasks for cell grouping detection, aiming to more effectively learn the specific structure of cells. Subsequently, we merge region proposals from grouping detection to achieve refined detection. In addition, according to the Bethesda system, clinical definitions among various categories of abnormal cervical cells are complex, and their boundaries are ambiguous. Differences in assessment criteria among pathologists result in ambiguously labeled cells, which poses a significant challenge for deep learning networks. To address this issue, we perform a labels correction module with feature similarity by constructing feature centers for typical cells in each category. Then, cells that are easily confused are mapped with these feature centers in order to update cells’ annotations. Accurate cell labeling greatly aids the classification head of the detection network. We conducted experimental validation on a public dataset of 7410 images and a private dataset of 13,526 images. The results indicate that our model outperforms the state-of-the-art cervical cell detection methods.

## 1. Introduction

Cervical cancer is one of the common malignant tumors that pose a significant threat to women’s health [1,2]. There has been a concerning trend towards younger age groups developing cervical cancer in recent years. Cervical cytology screening can detect early lesions, and early diagnosis and treatment can effectively reduce both the incidence and mortality rates of cervical cancer [3]. Traditional screening methods involve sampling and slide preparation followed by the microscopic examination of cellular morphology by pathologists [4,5]. However, interpreting cervical cytology slides can be quite challenging, requiring experienced pathologists for more accurate diagnoses [6]. Unfortunately, due to the variability in pathologists’ expertise and the unequal distribution of pathological healthcare resources, cervical cancer screening faces numerous difficulties and obstacles. This is especially true in resource-limited developing countries, where many patients are unable to be screened for precancerous lesions in a timely manner or may receive incorrect diagnosis, leading to increased challenges in subsequent treatment [7]. Therefore, the application of computer-aided pathological image analysis technology to enhance the accuracy and efficiency of diagnoses by physicians holds significant research value [8].

Pathological image analysis technology is a focal point of research in the field of digital healthcare. Computer-aided analysis models have become important tools for pathologists to use in diagnosing diseases, alleviating issues of missed or erroneous diagnoses resulting from subjective interpretation, and enhancing the diagnostic efficiency of physicians [9]. In recent years, an increasing number of researchers have applied machine learning and deep learning techniques to the analysis of cervical cytology images. Machine learning relies on a typical manual feature design [10], which requires researchers to possess adequate clinical knowledge about cervical cytology, including a thorough understanding of cellular shape, size, and color differences [11]. After feature extraction, further selection is necessary to improve model performance [12]. In contrast, deep learning networks begin from a random state and achieve optimal parameter conditions through a gradient learning method of backpropagation, ensuring the effectiveness of target feature extraction [13]. Pathological image analysis technology has demonstrated excellent performance in various medical tasks, including lesion detection [14], object segmentation [15,16], and classification [17]. Moreover, the interpretation of whole-slide images (WSIs) is a primary task in pathological image analysis, providing valuable references for pathologists’ diagnoses. However, due to the enormous pixel volume of WSIs, current computational technologies face challenges in processing these images in a single input. As a result, WSIs are typically divided into smaller image patches which are then classified as individual instances [18]. Alternatively, positive cells can be detected from these patches, and the identified positive cells can represent WSIs for classification purposes [19].

Cancer lesion and cancer cell detection are crucial steps in pathological image analysis [20,21]. Researchers have employed various models, including YOLO [22], faster R-CNN [23], dense-cascade R-CNN [24], and improved MACD R-CNN [25], for the detection of atypical cervical cells, achieving commendable performance. Liang et al. [26] proposed a global context-aware framework that integrates global context information through a classification branch and weighted loss. This approach combines the predictions from the branch into the cell detection process in order to filter out false-positive cells. Fei et al. [27] proposed a distillation framework that utilizes a pre-trained network to guide the training of detection networks. This framework can be applied to various detectors without altering their architecture during the inference process. Jiang et al. [28] introduced an interpretable method for detecting atypical cervical cells, encouraging the model to focus more on diagnostically significant abnormal cells and nuclei by incorporating a dual-stream self-attention module to enhance the learning of lesion-specific features. Wei et al. [29] integrated image-level classification into the cell detection network in order to filter out false-positive cells.

The aforementioned studies do not fully address the following issues. First, the distribution of cell categories in datasets is imbalanced. Second, medical data often come with significant clinical prior knowledge, leading to substantial visual differences among cells of various pathological types. Third, cell categories can easily be confused, and the inevitable noisy labels that arise during the labeling process can negatively impact the detection model. This paper uses existing detection models (such as YOLO or Faster R-CNN) as a backbone network and employs them as detectors for initial cell detection. Furthermore, we split the detection head into multiple groups in order to simultaneously detect various subtypes of cells. Subsequently, results from multiple group detections are recombined and merged into a single detection head for refined cell detection. The main contributions of this paper are summarized as follows:

- A new framework for detecting abnormal cells in pathological images has been proposed. This framework integrates cervical clinical prior knowledge and morphological similarities into the detection network to enhance performance in abnormal cell detection.
- A data augmentation module based on data frequency distribution has been introduced in order to achieve a balance in the number of different cell categories. This module adaptively adjusts the transformation methods based on the categories of cells in different datasets, aiming to improve the recall rate of difficult-to-detect categories.
- A cell grouping detection module based on clinical prior knowledge has been proposed. A traditional region proposal network is used to generate initial regions of interest containing all cell categories. Subsequently, the generated region proposals are grouped to calculate loss, optimizing the parameters of the fully connected layers corresponding to specific cell category detection.
- A confusing labels correction module based on feature similarity has been introduced. This includes constructing feature centers for each category and correcting cell labels by measuring the distance between detected cells and their corresponding feature centers.

## 2. Related Works

### 2.1. Criteria for Cervical Cells Interpretation

**Figure 1:**
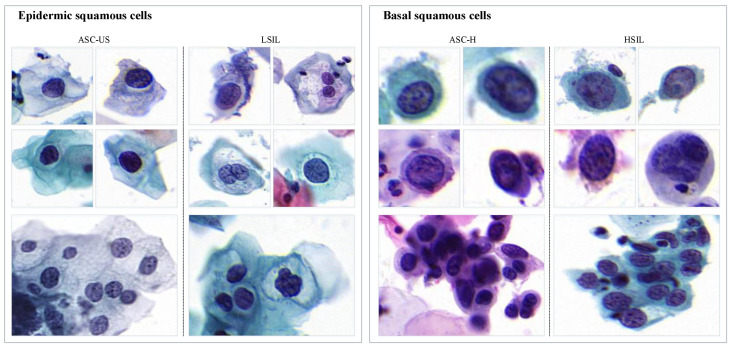
Examples of abnormal cervical squamous epithelial cells based on TBS. The first two rows represent individual cells, while the third row depicts cell clusters. Four categories of abnormal cells are classified into two groups: epidermic squamous cells and basal squamous cells.

The Bethesda system (TBS) is a commonly used standard for cervical cytology interpretation [30]. It classifies precancerous lesions of cervical squamous epithelial cells into four categories: low-grade squamous intraepithelial lesion (LSIL), atypical squamous cells of undetermined significance (ASC-US), high-grade squamous intraepithelial lesion (HSIL), and atypical squamous cells—cannot exclude HSIL (ASC-H). Cell examples are shown in Figure 1.

ASC-US and LSIL, which originate from epidermic squamous cells, typically appear in the early stages of HPV virus invasion and are associated with lower degrees of lesions. In contrast, ASC-H and HSIL, originating from basal squamous cells, are considered high-risk precancerous lesions. The characteristics of these two cell types show significant differences [31]. Based on this, we categorize precancerous cells into two subsets: epidermic cells and basal cells. Additionally, we observed that during the cell labeling process, some abnormal cells are clustered together, making them difficult to distinguish individually, requiring annotation at the cluster level. There are considerable morphological differences between cell clusters and individual cells. Therefore, we further divide the cell dataset into the other two subsets: cell clusters and individual cells.

According to TBS, there is no clear boundary between ASC-US and LSIL, making it easy for pathologists to confuse these two categories during the diagnostic process. Such confusion in diagnosis is tolerated to a certain extent [30]. Similarly, the same ambiguity exists between ASC-H and HSIL. However, for computer-aided analysis, this confusion can easily introduce intra-class noise labels. This represents an extremely unstable factor for the classification head in detection networks, significantly challenging the robustness of detection models.

### 2.2. Studies on Cervical Cells Detection

Many existing methods have conducted extensive research on the detection of cervical cells [32]. This paper primarily focuses on three issues related to detection: First, there is an imbalanced distribution of the number of cells in different categories. Zhang et al. [33] designed the unified strong and weak augment strategy method for image augmentation, setting a series of parameters to limit the number and extent of image transformations. However, image augmentation in that study was completely random and did not consider balancing the quantities of different cell categories. Our research addresses this by adaptively adjusting the probabilities of image transformations in order to balance the number of cells across categories. Second, this study aims to incorporate clinical prior knowledge into network learning. Guidance from clinical knowledge is crucial in medical-related research, often yielding higher accuracy with a lower learning cost. Chen et al. [34] classified annotated cells into single cells and clustered cells based on the morphological characteristics of cervical cells. Based on this foundation, our study introduces clinical diagnostic standards in order to further categorize the cells into epidermic cells and basal cells by their labels. Third, this study focuses on feature similarity analysis. In addition to the challenges of obtaining a large volume of medical images, a significant issue lies in the difficulty of labeling, particularly for cervical cells, where label confusion is common. Most studies tackle this problem using semi-supervised learning. Liang et al. [35] proposed a comparison detector that uses *K* samples to construct category prototypes and calculate the distances between instances and prototypes to correct confused samples. However, that research overlooks the diversity of cervical cells, as a limited number of *K* samples cannot adequately represent all cells of different categories. In our study, we adopt a memory bank to store the feature centers of categories and use momentum to update these feature centers in real time. This approach enables better adaptation to a diverse set of samples, addressing the issues of variability in cervical cell characteristics more effectively.

## 3. Methodology

### 3.1. Classification Framework

**Figure 2:**
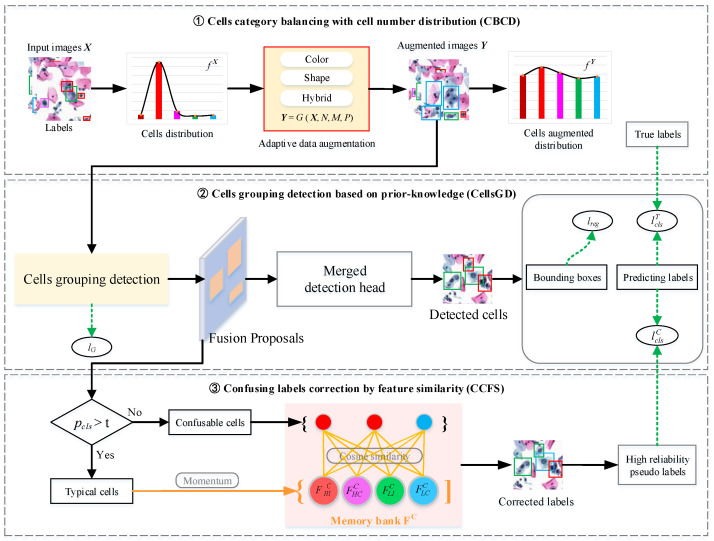
Cervical cell detection process based on prior knowledge grouping and the correction of confusing labels.

This paper proposes a cervical cell detection network based on prior knowledge grouping and the correction of confusing labels (PGCC-Net). The framework, as shown in Figure 2, comprises three modules: category balance with number distribution (CBCD), abnormal cell grouping detection based on prior knowledge (CellsGD), and the correction of confusing labels by feature similarity (CCFS) for loss optimization.

The category balance module utilizes data augmentation to balance the number of cells across different categories. Let all labeled data be represented as *X*, from which we calculate the frequency of cells with different categories, denoted as $f^{X}$. In a dataset, the distribution of cell numbers represented by $f^{X}$ is often imbalanced, which adversely affects subsequent detection and classification tasks. To alleviate this issue, we apply three common image augmentation methods: color transformation, shape transformation, and hybrid transformation. A set of parameters {*N, M, P*} is established to constrain transformation process *G* (∙). The augmented dataset, denoted as *Y*, is created, and the corresponding cell labels are also augmented, which serve as true labels for the detection model. The frequency of occurrence of labeled cells in *Y*, denoted as $f^{Y}$, will be relatively balanced, which is beneficial for subsequent cell detection tasks.

The cell detection module utilizes prior features for the grouping and prediction of abnormal cells. The detection process is divided into two phases. In the first phase, augmented images are passed through a convolutional neural network (CNN) to extract deep features. These features are then used to obtain original proposals according to predefined groupings, allowing for a preliminary detection of cells. Following this, results from the grouped detections are fused to obtain fusion proposals, which are subsequently fed into a merged detection head for the refined detection of anomalous cells. TBS serves as a diagnostic criterion for cervical cytology, and this module employs both TBS and morphological features as the basis for grouping.

Correction module for confusing labels utilizes cells identified during grouping detection to correct potential noisy labels which may have been generated during the annotation process. The classification head of grouping detection outputs both cells’ category and their corresponding classification probabilities. We classify cells with classification probabilities $p_{cls}>T$ as typical cells, while those with $p_{cls}<T$ are considered confused cells. Typical cells are then used to construct feature centers, and the similarity between each confused cell and feature centers is calculated. The category with the highest similarity serves as the corrected label for this cell.

### 3.2. Categories Balancing with Cells Number Distribution (CBCD)

Data augmentation involves applying various transformations to existing data in order to generate new training samples, thereby increasing the diversity and quantity of the dataset [36]. This approach effectively addresses the problem of data scarcity, helping to mitigate model overfitting and enhance the model’s generalizability [37]. In this paper, a substantial number of samples have been annotated for model training. However, during the annotation process, we observed that the distribution of cell number across different categories in the sample set was severely imbalanced. Therefore, the aim of the data augmentation in this section is not merely to increase the volume of data, but rather to balance the number of cells across different categories within the dataset.

SCAC Detector [33] builds upon RandAugment [38] and introduces improvements by incorporating three augmentation categories: color transformation, shape transformation, and hybrid transformation. A set of hyper parameters {*N, M, P*} is defined, where *N* represents the number of transformation methods used, *M* indicates the degree of augmentation, and *P* signifies the probability of applying a particular transformation method to an image. The transformation function is described by Formula (1):

(1) $$Y,L^{Y}=G(X,L^{X},N,\;M,P)$$

Here, *X* represents the input dataset, *Y* denotes the augmented dataset, and *G* (∙) is the data transformation function. Corresponding cell labels perform the same transformations, where ($L^{X},L^{Y}$) indicates cell labels before and after image augmentation. We calculate *P* using the statistical method in order to balance the distribution of cell number across different categories in the dataset.

At first, we establish image attributes $\left\{x,\;L,A^{x}\right\}$, where x∈X represents the input image. We count the largest number of cell labels in *x*, which is assigned as label L∈{1,2,…,n}. If cell counts of different categories in *x* are equal, then we select the category with a higher degree according to TBS as *L*. *n* represents the number of cell categories in the dataset. $A^{x}$ denotes the probability distribution of the transformations for each category in *x*, and the calculation process is as follows:

We compute the frequency distribution for different categories of cells in dataset *X*, denoted as $f^{X}=\{f_{1}^{X},f_{2}^{X},\ldots ,f_{n}^{X}\}$.

(2) $$f_{i}^{X}=\frac{N_{i}^{X}}{\sum_{i=1}^{n}N_{i}^{X}}$$

$N_{i}$ represents the cell number of *i*-th category in *X*. For categories with fewer cells, we hope to increase their transformation probability. Therefore, a power function is used to calculate the expected augmentation probability distribution $A^{X}$ for each category in *X.*

(3) $$A_{i}^{X}={\left(f_{i}^{X}\right)}^{-1}$$

Similarly, we compute the frequency distribution for different categories of cells in image *x*, denoted as $f^{x}=\{f_{1}^{x},f_{2}^{x},\ldots ,f_{n}^{x}\}$.

(4) $$f_{i}^{x}=\frac{N_{i}^{x}}{b+\sum_{i=1}^{n}N_{i}^{x}}$$

*b* is a small constant used to avoid the issue of having a denominator equal to 0 in the formula when there are no abnormal cells present in image *x*. Subsequently, we compute the Hadamard product between the two distributions mentioned above, described by Formula (5). Then, result is normalized using SoftMax [39] to obtain probability distribution $A^{x}$.

(5) $$f_{i}=A_{i}^{X}⊙f_{i}^{x},\;i\in \{1,2,\ldots ,n\}$$

(6) $$A_{i}^{x}=Softmax\left(f_{i}\right)=\frac{e^{f_{i}}}{\sum_{i=1}^{n}e^{f_{i}}}$$

SoftMax maps output values to the range [0, 1] and constrains the sum of output values to equal 1. After that, based on image label *L*, we select $A_{L}^{x}$ as the transformation probability *P* for image *x*. SoftMax will amplify the distance between values, which is usually the desired outcome. However, in order to prevent the transformation probabilities from becoming too extreme (close to 0 or 1), we set a minimum transformation probability of 0.2 and a maximum transformation probability of 0.8.

(7) $$P=\left\{\begin{array}{cc} 0.2, & P\leq 0.2\\ P, & 0.2<P<0.8\\ 0.8, & P\geq 0.8 \end{array}\right.$$

In experiments, we found that different transformation methods have varying effects on the recall of each category. In this study, three transformations are employed: color, shape, and hybrid. Therefore, weight factors {α,β,γ} are set to adjust the probabilities of applying different types of transformations. Based on Reference [33], the transformation parameters used in this study are shown in Table 1.

For labeled input image *x*, transformations are selected based on weight factors {α,β,γ}. Then, the CBCD module randomly chooses *N* methods, for example mix-up, from the corresponding transformation and performs the transformation operations according to the probability *P*. Transformation magnitude *M* lies within the range of [0.5, 1.0]. Weight factors {α,β,γ} are adaptively adjusted according to the category, which will be detailed in Section 5.2.

### 3.3. Cell Grouping Detection Based on Prior Knowledge (CellsGD)

#### 3.3.1. Grouping with Prior Knowledge

**Figure 3:**
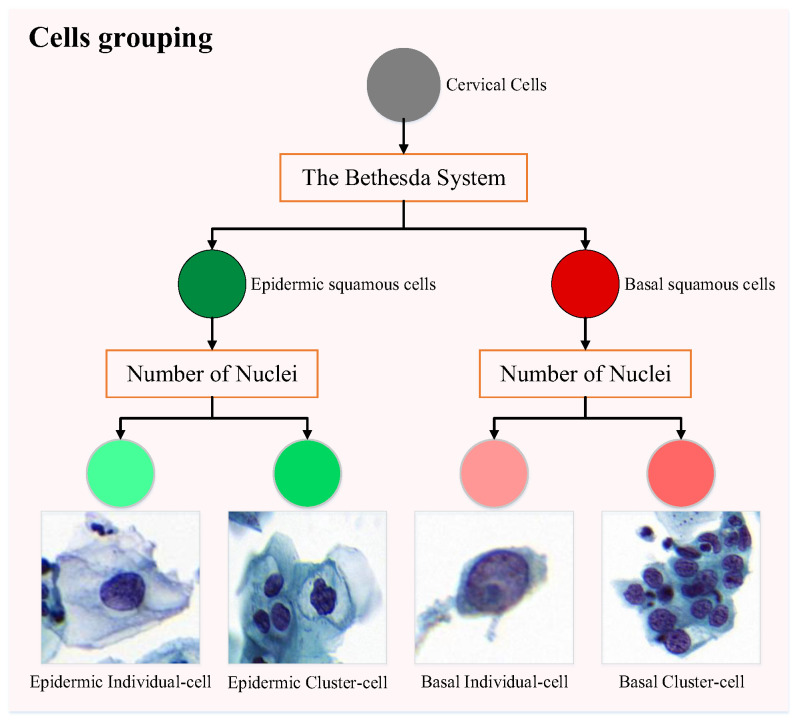
Grouping process of cervical precancerous lesion cells.

Pathologists annotate cervical precancerous lesion cells into four categories: {HSIL, ASC-H, LSIL, ASC-US}, where {HSIL, ASC-H} belong to basal squamous cells, and {LSIL, ASC-US} belong to epidermic squamous cells. These two categories exhibit significant morphological differences. According to TBS, there is no clear boundary between ASC-US and LSIL, which often leads to confusion during the diagnostic process by pathologists. Similarly, a comparable interpretation exists between ASC-H and HSIL. Therefore, we classify basal squamous cells and epidermic squamous cells into different groups. In addition, it is often challenging to distinguish the boundaries of cells in clusters, whereas cell nuclei are relatively easier to identify. We utilize a nuclear detection method [23] to obtain the number of cells within each annotated box to divide individual cells and cell clusters. Subsequently, we manually correct the partitioning results to achieve more accurate groupings. The process is illustrated in Figure 3.

Hence, cervical annotations are reclassified into four types of annotations {EI: epidermic individual cell, EC: epidermic cluster cell, BI: basal individual cell, BC: basal cluster cell}.

#### 3.3.2. Abnormal Cell Detection

**Figure 4:**
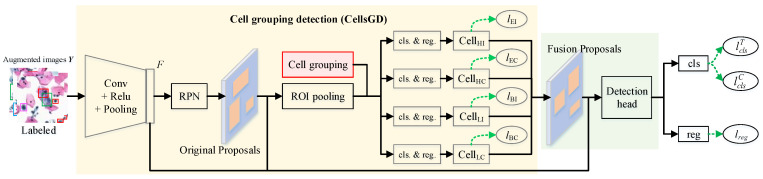
Grouping detection process of cervical precancerous lesion cells.

A two-stage cell detection method is employed in cell detection: grouping detection with prior knowledge and proposal merging for optimization detection. In the first stage, a traditional region proposal network (RPN) [40] is used to generate original regions of interest, and the generated region proposals are grouped for training to obtain preliminary detection results for different groups of cells. In the second stage, detection results from each group are merged to produce fusion proposals, which are then refined through ROI pooling and fully connected layers for detecting abnormal cells. The process is illustrated in Figure 4.

Specifically, the data with balanced cell categories are input into CNN, passing through multiple network layers (Conv + ReLU + Pooling) in order to obtain deep image features (*F*). *F* serves as the input for the subsequent RPN and ROI pooling. RPN uses SoftMax to classify anchors as positive or negative and then applies bounding box regression to refine anchors, generating the proposals for the first stage. After collecting *F* and proposals, RoIPooling extracts proposal features and forwards them to the following fully connected layers to determine target classes. We modify the traditional object detection framework by dividing detection targets into four groups {EI, EC, BI, BC}. Proposal features are fed into four separate fully connected layer branches for classification prediction and bounding box regression, resulting in the detection of different grouped cells {Cell_EI_, Cell_EC_, Cell_BI_, Cell_BC_}. Each branch has the same fully connected layer structure, but parameters are not shared. Grouping detection loss is defined as $l_{G}=l_{EI}+l_{EC}+l_{BI}+l_{BC}$. At this point, we obtain the results of cell group detection. In the second stage, {Cell_EI_, Cell_EC_, Cell_BI_, Cell_BC_} are merged into a new region proposal, which is then input into the detection head for refining the detection of abnormal cells. In this stage, in addition to the regression loss $l_{reg}$, two classification losses are computed: loss by true labels $l_{cls}^{T}$ and loss by corrected labels $l_{cls}^{C}$.

### 3.4. Confusing Labels Correction by Feature Similarity (CCFS)

Due to the fuzzy boundaries between different categories of cervical cells, diagnostic standards among pathologists cannot be fully unified, leading to inevitable noisy labels within the dataset. The loss of detection model consists of two components: classification loss $l_{cls}$ and regression loss $l_{reg}$. The primary aim of classification loss is to accurately identify detected targets, while regression loss aims to achieve better bounding boxes for detection. The presence of noisy labels has a significant impact on the classification loss. To address this issue, we propose a correction module for confusing labels with feature similarity in order to optimize the loss in the classification head. This module aims to enhance the accuracy of classification by adjusting labels associated with detected cells, thereby reducing the detrimental effects of noisy labels and improving the overall performance of the detection model.

From the perspective of model learning, noisy samples often exhibit lower typicality in their annotated categories, meaning that they have lower classification probabilities. We denote cells with classification probabilities above threshold T as typical cells, and those with classification probabilities below T as confusing cells. The incorrectly labeled confusing cells are considered noisy samples. Based on this, we calculate feature centers for each category $\left\{F_{EI}^{C},F_{EC}^{C},F_{BI}^{C},F_{BC}^{C}\right\}$ using typical cells. Since not all images contain sufficient typical cells, we design a memory bank $F^{C}=\{F_{EI}^{C},F_{EC}^{C},F_{BI}^{C},F_{BC}^{C}\}$ to store feature centers.

Feature centers are initialized using the mean CNN features of labeled typical cells. Subsequently, momentum [41] is employed to update $F^{C}$. For instance, taking the feature center of epidermic individual cells $F_{EI}^{C}$, we select cell features with a probability above T and belonging to the EI category from fusion proposals during grouping detection, and we update the feature center using Formula (8):

(8) $$\begin{array}{c} F_{EI}^{C}=\tau F_{EI}^{C}+\frac{\left(1-\tau \right)}{m}\sum_{h=1}^{m}F_{h},h\in \left\{1,2,\ldots ,\;m\right\}\\ \tau =\frac{1}{e+1} \end{array}$$

$F_{h}$ represents the *h*-th typical EI cell among *m* typical cells. *e* denotes the number of iterations, and *τ* is the momentum coefficient. *τ* decreases as the number of iterations increases, which means that in early stages of training, the update rate of $F^{C}$ is relatively high, and it gradually becomes smaller as the number of iterations increases.

Subsequently, we extract features of confusing cells output by grouping detection, denoted as $\left\{F_{1},\;F_{2},\;\ldots ,\;F_{r}\right\}$, where *r* represents the number of confusing cells outputted by the detection model. Then, cosine similarity (*S*) is calculated between cell features (*F*) and feature centers ($F^{C}$).

(9) $$S_{ij}=\cos\left(F_{j},F_{i}^{C}\right),j\in \left\{1,2,\ldots ,\;r\right\},i\in \left\{1,2,\ldots ,\;n\right\}$$

$S_{ij}$ represents the similarity between the *j*-th confusing cell and the *i*-th category. The category corresponding to the feature center with the highest similarity ($max(S_{ij})$) is considered the corrected label for this confusing cell.

Corrected labels and true labels are used together to compute classification loss $l_{cls}$ for the detection model. The final detection loss is the sum of classification loss and detection loss.

(10) $$l_{cls}=l_{cls}^{T}+{\lambda l}_{cls}^{C}$$

(11) $$loss=\frac{1}{r+m}\sum_{k=1}^{r+m}{(l}_{cls}(x_{k})+l_{reg}(x_{k}))$$

$l_{cls}^{T}$ represents the classification loss associated with true labels, while $l_{cls}^{C}$ denotes classification loss based on corrected labels. λ controls the contribution of corrected labels. In addition, $l_{cls}$ is computed using cross-entropy loss, and $l_{reg}$ is computed using *L*_1_ loss. $x_{k}$ represents the *k*-th detected cell in image *x*.

## 4. Experiments and Results

### 4.1. Datasets

This paper uses two sets of data for experimentation. The first set is a private dataset, with liquid-based cytology images of cervical cells obtained from multiple hospitals. Whole slide images (WSIs) were acquired using the same scanner to produce 20× images. A total of 13,526 images were cropped from WSIs for annotation. Special thanks to five pathologists for their contributions to the data accumulation for this study. All images were annotated at the cellular level by three pathologists and were subsequently reviewed by a pathologist with over ten years of diagnostic experience. For any disputed annotations, discussions were held under the leadership of another pathology expert to reach a consensus. A total of 98,355 cervical abnormal cells were annotated, including four categories {HSIL, ASC-H, LSIL, ASC-US}.

The second set is a public dataset, called ComparisonDetector [35]. WSIs were obtained using the Pannoramic MIDI II digital slide scanner, and corresponding specimens were prepared using the Thinprep method and stained with Papanicolaou stain. A total of 7410 images were cropped from WSIs, resulting in 48,587 annotated cells across 11 categories {ASC-US, ASC-H, LSIL, HSIL, SCC, atypical glandular cells, trichomonas, candida, flora, herpes, actinomyces}. Since this study primarily focuses on the detection of squamous epithelial cells, categories {HSIL, ASC-H, LSIL, ASC-US} were selected for validation. Additionally, squamous cell carcinoma (SCC) is not classified as a precancerous lesion, morphological characteristic of which are similar to HSIL. Therefore, we categorized it into the basal cells group.

**Figure 5:**
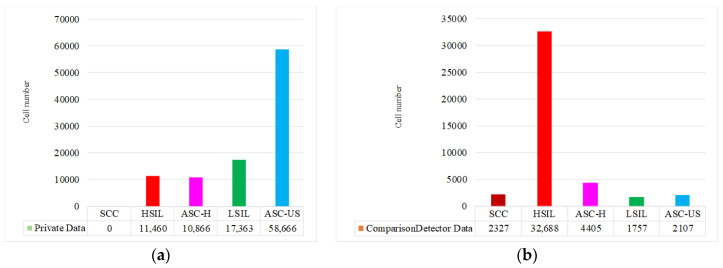
Distribution of cell numbers in each category. (**a**) Private dataset; (**b**) ComparisonDetector public dataset.

Figure 5 shows histograms of data distribution for private dataset and ComparisonDetector dataset.

Both private and public datasets exhibit imbalanced distributions of cell numbers. Interestingly, the distribution of cell numbers across categories significantly varies between two datasets. In the private dataset, the number of ASC-US cells is relatively high, which is consistent with the proportion of cases in clinical interpretations. In contrast, the ComparisonDetector dataset contains an unusually high number of HSIL cells. This discrepancy may be due to researchers intentionally selecting images with high-grade lesions for cellular-level annotation in order to enhance the detection of HSIL cells. During the experimental phase of this study, training and validation sets were divided in a ratio of 8:2.

In the process of analyzing cervical cytology images, models focus more on the morphological features of targets. Data augmentation methods used in this paper maintain the morphological invariance of cells, such as color transformations, rotations, and channel transformations. Therefore, cell labels in images generated in this paper will not change. In addition, we specifically invited two pathologists to review the images after data augmentation to ensure the accuracy of cell annotations. Table 2 shows the number of cells in each category before and after data augmentation of the training set.

From Table 2, it can be seen that the datasets still exhibit imbalance in terms of cell quantities after data augmentation. This issue is particularly pronounced in the ComparisonDetector dataset, where the significant disparity in the number of original samples prevented augmentation methods from completely resolving this problem. However, the contribution of augmentation methods is still noteworthy. For instance, in the ComparisonDetector dataset, we reduced the ratio of HSIL to ASC-H from approximately 7:1 to 2:1, which has alleviated the issue of severe sample imbalance.

### 4.2. Experimental Setup and Evaluation Criteria

During the training process, a pre-trained Faster R-CNN is utilized for cell grouping detection (CellsGD). Then, fixing the parameters of the CellsGD network, subsequent detection tasks are performed. We train models with an initial learning rate of 0.005 and 50 iterations. Additionally, the parameter b used in CBCD is set to 0.0001, a small constant. Threshold T used in the CCFS module is set to 0.95, a value determined by three pathologists based on the typicality of cells detected in the CellsGD stage. λ=0.3 is obtained through multiple iterative experiments. The architecture of the CellsGD in the experiment is shown in Table 3.

The CellsGD architecture shown in Table 3 consists of four components. The Faster R-CNN encoder is used to extract deep feature maps from images, and then feature maps are input into the RPN encoder to obtain proposals. After that, proposals are processed through grouped and fusion detection heads to yield the final detection targets. *N* denotes the number of groups, and *k* represents the final number of detected cells.

This study conducts ablation experiments and comparative experiments to validate the performance of the proposed method. Ablation experiments specifically assess the effectiveness of the cell category balance module (CBCD), cell group detection module (CellsGD), and label correction module (CCFS). Comparative experiments involve benchmarking the proposed PGCC-Net against existing detection networks. In experiments, the performance of the detection methods is evaluated using average precision (AP) and average recall (AR) [42]. AP is the mean precision score calculated at various intersection over union (IoU) thresholds, ranging from 0.5 to 0.95 with a step size of 0.05. The experimental environment is established using the PyTorch 1.10.0 library and Python 3.7, with training conducted on a workstation with a single RTX 3090 GPU. Based on the current operating environment, we have calculated the training and inference time for each module. The results are shown in Table 4.

As shown in Table 4, both the CBCD module and CCFS module utilize traditional image processing methods and do not involve iterative learning through networks. Therefore, the primary influencing factor is the I/O speed of the computer. The average training time for the CellsGD module, which may be affected by other tasks, was calculated over multiple training runs to be 10 h. During the inference phase, only the CellsGD module was utilized, which is based on Faster R-CNN, achieving an inference time of 128 ms on each patch.

### 4.3. Ablation Experiment

We conducted ablation experiments on both the private dataset and the ComparisonDetector dataset in order to evaluate the effectiveness of the various proposed modules, including CBCD, CellsGD, and CCFS. Table 5 reports the AP and AR for cell detection using different modules across two datasets. To facilitate the description of differences between the experimental groups, we have named the eight groups of experiments A to H. Each group used a different combination of three modules.

As shown in Table 5, the ablation experiments were set up with eight groups. Group A utilizes Faster R-CNN as a baseline detection network. Groups B to G present experimental results for various combinations of the proposed modules. Group H represents the proposed PGCC-Net, which achieved the highest detection metrics across both datasets compared to the other experimental groups, with an AP of 34.3 and 33.8 and an AR of 57.1 and 56.6. Next, we will perform a module-wise analysis of the results from the ablation experiments.

The CBCD module utilizes the distribution of cell numbers across different categories to augment datasets. Results in group B, which solely incorporates the CBCD module, show a slight improvement in AP compared to the Faster R-CNN (group A). However, both datasets experience a decrease in AR. A similar trend is observed in group F as well. This situation is closely related to the severe class imbalance present in the datasets. As described in Section 4.1, there is an excess of ASC-US cells in one dataset and an abundance of HSIL cells in the other. After data augmentation, the overall detection accuracy for targets improves. However, when calculating the AR metric, results tend to be biased towards more abundant cell categories. To further validate the effectiveness of CBCD, we calculated detection metrics for each category. As shown in Table 6, QR represents the proportion of each category in the dataset.

Due to the lack of annotated SCC in the private dataset, we are e unable to calculate detection metrics for this category. As shown in Table 6, the AR for ASC-US in the private dataset has slightly decreased, as the balanced dataset imposes certain limitations on detection of ASC-US. Similarly, the AR of HSIL in the ComparisonDetector dataset has also decreased. In contrast, the AR of LSIL and ASC-H have increased with data augmentation. Overall, the AP for each category has seen a slight improvement across both datasets, indicating that the CBCD module plays a beneficial role in detecting categories with few samples of cervical cells.

The CellsGD module divides cell detection into four sub-tasks based on morphological characteristics and TBS as prior knowledge. Compared to Faster R-CNN (group A), the results of group C show a significant improvement in AP on both datasets, reaching 32.3 and 31.7. There is also a slight increase in AR. Interestingly, group E demonstrates that reduced AR caused by the CBCD is compensated for by CellsGD, as groups containing categories with few samples are not affected by quantity during grouped detection. Overall, the ablation study results reveal that CellsGD contributes the most to the model designed in this paper. We will discuss grouping results in detail in Section 5.3.

The CCFS module corrects cells’ labels by calculating the similarity between features of confusable cells and the feature centers of the category, thereby increasing the accuracy of the classification head in the detection network. Both the AP and AR for group D are superior to Faster R-CNN (group A). Group G validates the integration of CBCD and CCFS, yielding a slight improvement. Unfortunately, CCFS did not significantly enhance detection AR. Although the overall performance of detection improved, metric calculations still tend to focus on categories with a large number of samples. In group F, the AP of combined CellsGD and CCFS reached 33.9 and 33.3, which is only slightly different from the results of PGCC-Net (group H).

### 4.4. Comparative Experiments

**Figure 6:**
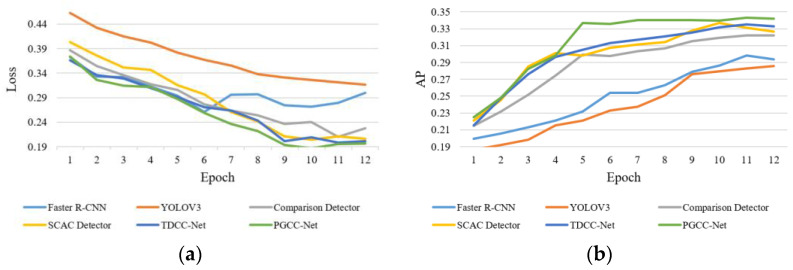
Variations of training loss and AP on six methods. (**a**) Loss variation of different methods; (**b**) AP variation of different methods.

We compare the performance of PGCC-Net with existing cervical cell detection methods, including Faster R-CNN [40], YOLOv3 [43], ComparisonDetector [35], SCAC Detector [33], and TDCC-Net [34]. Faster R-CNN and the YOLO series are mainstream two-stage and one-stage detection networks. ComparisonDetector utilizes a proposal-based object detection approach, replacing the category of each proposal by comparing its classification with prototype representations of each category. It also provides a public dataset used in this study. SCAC Detector devised a unified strong–weak enhancement strategy to increase the diversity of training data and employs a transformer as a backbone to capture long-range dependencies. TDCC-Net breaks down the original detection task into two sub-tasks and models them separately in order to learn more effective and useful feature representations for specific cellular structures. Additionally, this network uses a dynamic comparison module and instance contrastive loss for cell comparison, mimicking expert clinical diagnosis. Figure 6 shows line charts of training loss and AP variations for the above six methods in the private dataset.

Since most of the methods in comparative experiments use Faster R-CNN as a backbone network, we set the training epochs to 12. Except for YOLOv3, the other methods tended to converge. Figure 6a shows the losses change curve, where Faster R-CNN begins to converge by the 6th iteration, and the other methods converge by the 9th iteration. Figure 6b presents the AP change curve, where we can see that our proposed PGCC achieves the best detection performance. Table 7 lists the comparative experimental results on both datasets.

From Table 7, it can be observed that among existing detectors, Faster R-CNN outperforms YOLOv3 in terms of detection. Therefore, this study selects Faster R-CNN as the backbone network. Comparison Detector shows a significant improvement in detection compared to Faster R-CNN and YOLOv3, particularly in terms of AP on the private dataset, which increases by 2.4 and 3.6, respectively. This indicates that similarities between cells contribute positively to the classification head of the detection network. SCAC Detector demonstrates better performance, and the establishment of global associations through transformers is a current research hotspot, proving to be highly beneficial for integrating the context information of targets. It is noteworthy that, as described in Section 4.1, the private dataset contains a larger number of superficial cells, while the ComparisonDetector dataset has a higher quantity of basal cells. Morphological differences between these two types of cells can lead to a certain bias in the learning detection network. SCAC Detector shows smaller performance discrepancies across two datasets compared to other detection networks, indicating that the data enhancement strategy proposed by SCAC can improve the stability of model training. TDCC-Net enhances cell comparison analysis by incorporating grouped detection, resulting in improved detection performance compared to ComparisonDetector, which validates that leveraging morphological prior knowledge through grouping can indeed benefit the performance of the detection network.

PGCC-Net demonstrates outstanding performance on both datasets. Compared to the backbone network Faster R-CNN, PGCC-Net achieves an improvement of 4.5 and 4.9 in terms of AP and an increase of 1.9 and 2.2 in terms of AR across the two datasets, respectively. In comparison to other methods, PGCC-Net achieves the highest values of AP and AR. PGCC-Net incorporates three modules focusing on balancing cell categories, grouping based on prior knowledge, and comparing cell similarity, respectively. This approach results in higher sensitivity and robustness in the recognition of cervical abnormal cells. Therefore, the method proposed in this paper is particularly suited for cervical cytological detection.

**Figure 7:**
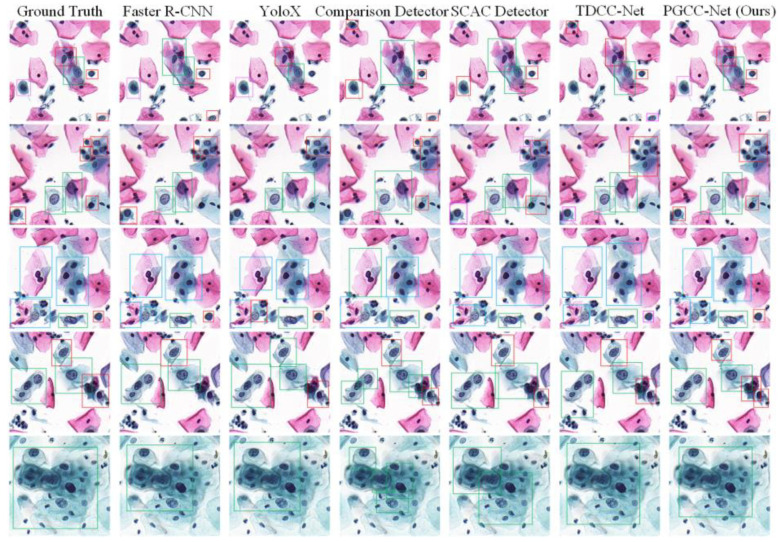
Qualitative comparison of different cervical lesion detection networks.

In order to qualitatively compare the performance of various detection methods, we visualize the results of six detection networks. In Figure 7, different colored boxes represent different cell category labels {Red: HSIL, Purple: ASC-H, Green: LSIL, Blue: ASC-US}. The first column displays the ground truth, while the remaining columns show the results from each detection network.

For better visualization, we cut out regions of interest measuring 500 × 500 pixels. From Figure 7, it is evident that both Faster R-CNN and YOLOv3 demonstrate good performance but still experience some false positives and missed detections. ComparisonDetector achieves high accuracy in detecting cells but shows lower robustness in distinguishing clustered and individual cells. SCAC Detector encounters a number of classification errors, which is related to the randomly augmented data it utilizes. TDCC-Net effectively differentiates between clustered and individual cells, although it also presents some false positives and missed detections. The proposed PGCC-Net delivers commendable results in both detection and classification. While there are instances in the second row of images where multiple cells are detected as a cluster, this does not affect the overall interpretation of images.

### 4.5. McNemar’s Test

McNemar’s test [44] is used to compare the statistical differences of classifiers in binary classification. It is based on cross-tabulation of misclassifications, where the observed frequencies of mismatched errors are computed and tested using binomial distribution. In order to reject the null hypothesis of two models being similar, the *p*-value of the test should be smaller than 0.05. We calculate the *p*-values of our study compared to existing studies separately on the private dataset set and the ComparisonDetector dataset. Results are shown in Table 8.

As shown in Table 8, we calculated the *p*-values of the proposed method and existing studies, and the results were all below 0.05. Therefore, the null hypothesis can be rejected, indicating a significant difference between our proposed method and existing detection methods.

## 5. Discussion

### 5.1. Overview

Pathological image cell detection technology mitigates the workload of pathologists and minimizes the issues of misdiagnoses. This paper proposes PGCC-Net for the detection of abnormal cervical cells and their classification according to TBS, achieving notable results. The method consists of three modules: a cell category balance module (CBCD), a cell group detection module (CellsGD), and a label correction module (CCFS). CBCD focuses on balancing the number of different cell categories, and through ablation studies, we found that this module significantly aids in the detection of minority classes. CellsGD stands out in our research contributions, as it utilizes prior knowledge for grouping. The sub-detectors concentrate more on the morphological features of cells within the group while minimizing interference from inter-group variations. As cervical cytological diagnoses depend on differences in cell morphology, the boundaries between categories following TBS can be ambiguous, often leading to mislabeling issues. The CCFS module adaptively corrects the labels of confused cells based on feature similarity, enhancing the stability of the classification head in the detection network. PGCC-Net cleverly integrates these three modules to improve both the accuracy and recall of cell detection.

### 5.2. Analysis of Images Augmentation

Image augmentation is commonly employed to increase sample size and improve the generalization performance of models. In this study, we utilize dynamic images augmentation to balance the quantity of cells in different categories. During our experiments, we observed that various image augmentation techniques have differing impacts on detectors. Using the ComparisonDetector dataset, we validated different augmentation methods. The results presented in Table 9 reflect the detector performance after image augmentation, using Faster R-CNN for learning. Here, AP_50_ represents average precision with a fixed IoU threshold of 50%.

In this study, three types of image transformations are employed: color (hue, saturation, brightness), shape (flip, scale, translate), and hybrid (Cutout [45], Mixup [46]). In Table 9, bolded values indicate the highest metrics, while underlined values represent the second highest metrics. From the results, it is evident that solely using color transformations for the augmentation of pathological images may actually lead to a decrease in detection accuracy. In contrast, the application of shape and hybrid transformations shows a significant improvement in detection performance, particularly with hybrid transformations contributing substantially to recall of cells. When all three transformations are utilized together, the overall performance of the detection network is notably enhanced.

Additionally, different types of transformations also impact the recognition of various cell categories. In Section 3.2, we established weight factors {α,β,γ} to adjust the probabilities of applying different transformations on images. The size of these weights is dynamically adjusted based on dataset characteristics. Table 10 and Table 11 present the performance of different transformations on cell detection across various categories in the ComparisonDetector dataset.

In the tables, bold values represent the highest metrics of respective columns, while underlined values indicate the second highest metrics. From Table 10, it is evident that using color augmentation alone results in suboptimal detection performance. Shape and hybrid methods contribute positively to improving detection accuracy, and the integration of three augmentations yields the highest detection precision. Table 11 illustrates that different transformations significantly affect recall for various cell categories, particularly for HSIL, where recall substantially decreases after image augmentation. This observation further corroborates the findings from our ablation study in Section 4.3. It is important to note that these results are based on the ComparisonDetector dataset, which exhibits a serious imbalance in category distribution, as shown in Figure 5b. We plan to address this issue in future research and continue to validate the impact of image transformations on detection performance.

We adaptively adjusted the image transformations utilizing parameters {α,β,γ} based on their corresponding detection performance of the dataset. As the analysis of Table 10 and Table 11, we set the weight factors to {α=0.1,β=0.4,γ=0.5} with the growth rates of contribution associated with each transformation. Future work will focus on developing a more systematic approach to parameter selection that better aligns with detection outcomes across various datasets. This ongoing research aims to refine the effectiveness of image augmentation strategies in enhancing model performance.

### 5.3. Analysis of Cells Grouping Detection

Deep learning techniques for computer vision primarily focus on the morphological features of targets, enabling the detection of subtle differences between them. In medical image analysis, guidance from clinical prior knowledge is crucial, as it allows for the division of subtasks based on the diagnostic habits of physicians. This approach ensures that subtasks do not interfere with one another, thereby alleviating the learning burden of the network. In this study, we categorized cells into two groups {individual cells, cell clusters} based on their morphological characteristics. Subsequently, guided by clinical prior knowledge (TBS standard), we further divided these two groups into epidermal cells and basal cells, resulting in a total of four sub-datasets. To validate the effectiveness of the grouping, we conducted three experiments on the ComparisonDetector dataset. The results of these experiments are presented in Table 12. We named the three groups of experiments I, J, and K. Each group represents a different multi-class experiment.

In Table 12, group I represents the detection of five classes using Faster R-CNN, according to TBS standards. Group J demonstrates the results obtained from dividing the dataset into epidermal cells and basal cells for the detection of two classes, showing improvements in AP and AR. Group K involves the detection of individual cells and cell clusters, as two-class detection, and performs even better than the previous two groups. This indicates that the grouping based on morphological features is more advantageous for cervical cell detection. The ablation study in Section 4.3 also revealed that grouped detection significantly contributes to cell detection. Given the various regularities observed in cytological interpretation, there is potential to establish more groupings based on prior knowledge. In future research, we aim to explore more rational and diverse groupings in order to further enhance detection performance.

### 5.4. Granular Analysis of Cervical Cells Detection

To further analyze the detection capability of our model, we introduced three metrics, true positives (TP), false positives (FP), and false negatives (FN), to evaluate its performance. Since the two datasets used in this study did not include comprehensive annotations on negative cells, the true negative (TN) metric could not be calculated. The number of diseased cells tested in the two datasets was 19,671 and 8657, respectively. Table 13 presents the TP, FP, and FN values for different detection networks, where an upward arrow indicates that a higher value is better, while a downward arrow indicates that a lower value is preferable.

It can be seen from Table 13 that detection networks have different capabilities on the two datasets. The proportion of FP is higher on the private dataset than on the ComparisonDetector dataset, probably due to the fact that the private dataset contains more ASC-US cells, and this type of cell has a high similarity with negative cells. This causes networks to mislearn some negative cells as diseased cells. The impact of different datasets on network training will be an important direction for our future research.

### 5.5. Limitations and Future Works

Although our proposed method has improved the performance of cervical cell detection, it also has certain limitations. First, missed detections and false positives are two of the most common problems in detection models. Existing studies, including the framework presented in this paper, have contributed to improvements in detection accuracy. However, resolving these issues will require a sustained technical effort. In our next steps, we will continue to prioritize finding effective solutions to the problems of missed detections and false positives, which is a key focus of our research. Second, although we employed data augmentation methods to mitigate the sample imbalance in the datasets, this study does not fundamentally resolve this issue. For instance, our private dataset still shows a tendency of ASC-US detection, and the model trained on the ComparisonDetector dataset exhibits better detection performance for HSIL compared to other categories. To address this problem, one approach is to accumulate a larger volume of data, ensuring that various cell types are not only abundant but also balanced. Additionally, we plan to conduct a more in-depth study of the morphological differences among cells and explore more reasonable and diverse data augmentation strategies in future research in order to enhance the robustness of model training.

In future work, we will focus on optimizing our method’s structure in order to address these issues. Furthermore, our approach has the potential to be applied in other areas of cytopathology or histopathology images. In cytological images, morphological differences are the primary characteristics of cell subtypes. For instance, follicular tumors exhibit distinct structures compared to other subtype lesions and benign conditions in thyroid aspirate cells. This observation offers a direction for grouping. In histopathology images, differences between various tissue cells can be quite significant. We can first detect individual cells within tissue images and then combine them into different tissue regions. We will extend the applicability of this study to a broader range of detection. By doing so, we aim to enhance the utility and effectiveness of our approach in various contexts within medical image analysis.

Additionally, pathological images are relatively large, even exceeding 6000 × 6000 pixels. This magnitude of images surpasses the processing capability of computers. We use a sliding window approach to segment the images, with each image patch sized at 2048 × 2048 pixels. Sliding windows’ overlap is set to 128 pixels in order to avoid splitting positive cells in two patches. Subsequently, divided patches are resized to 512 × 512 pixels before being input into the detection model. Afterward, we will provide the detected cells to pathologists for assistance in the interpretation of whole slide images. Next, we will continue to accumulate different types of pathological data with the aim of building a more extensive database for deep learning research. Additionally, we will conduct in-depth studies on whole slide images in order to develop more efficient analysis models suitable for high-resolution images.

## 6. Conclusions

In this paper, we propose a cervical cell detection network called PGCC-Net, which is based on prior knowledge grouping and the correction of confusing labels for cervical lesion images. Specifically, we adaptively augment the dataset based on distribution of the number of different types of cells in order to achieve balance between the categories. Second, we break down the detection model into multiple sub-tasks using prior knowledge for cell grouping detection, aiming to learn more features of specific cells. The region proposals from grouping detection are then merged to achieve refined detection. Additionally, according to the Bethesda system, the clinical definitions of various categories of cervical abnormal cells are complex, leading to discrepancies in the diagnostic standards among pathologists. This results in some labeled data being easily confusable, posing a significant challenge for deep learning interpretations. To mitigate this issue, we perform label correction based on feature similarity. By constructing feature centers for typical cells in each category, confusable cells are mapped to these feature centers. Then, cell annotations are updated accordingly. Accurate cell labeling greatly aids the classification head of the detection network. Experimental results demonstrate that our proposed method exhibits superior detection performance on cervical cells.

## Figures and Tables

**Table 1:** Parameters applied in CBCD module.

Transformation	Weights	*N*	*P*	*M*_min_	*M*_max_
Color transformations	α	2	0.2 ≤ *P* ≤ 0.8	0.5	1.0
Shape transformations	β	2	0.2 ≤ *P* ≤ 0.8	0.5	1.0
Hybrid transformations.	γ	1	0.2 ≤ *P* ≤ 0.8	0.5	1.0

**Table 2:** The number of cells of each type in the training set.

Dataset	Augmentation	SCC	HSIL	ASC-H	LSIL	ASC-US
Private	Before	0	9168	8692	13,890	46,933
	After	0	48,297	46,333	5120	58,810
ComparisonDetector [35]	Before	1862	26,150	3524	1406	1686
	After	14,762	38,092	17,203	10,858	13,465

**Table 3:** Architecture of the CellsGD module.

CellsGD	Layers	Output
Input	/	Feature size: 512 × 512
Faster Rcnn encoder	Conv × 13 Relu × 13 Pooling × 4	Feature size: 256 × 256
RPN encoder	3 × 3 Conv	Proposal size: 7 × 7 × 512
Groups detection head	(1 × 1 Conv) × *N*	ROI: *N* groups
Fusion detection head	1 × 1 Conv	ROI: 1 groups Scores: 2*k* Coordinates: 4*k*

**Table 4:** Training and inference time of different modules.

**Modules**	CBCD	CellsGD	CCFS
Train time	≈1 h	≈10 h	30 min
Inference time	/	128 ms	/

**Table 5:** Ablation experiments for the CBCD, CellsGD, and CCFS modules.

Groups	Modules			Private Data		ComparisonDetector Data	
	CBCD	CellsGD	CCFS	AP	AR	AP	AR
A				29.8	55.2	28.9	54.4
B	√			30.2	54.6	29.5	54.1
C		√		32.3	55.3	31.7	55.1
D			√	30.7	55.7	30.2	54.8
E	√	√		32.5	55.5	32.1	55.2
F		√	√	33.9	56.7	33.3	55.1
G	√		√	31.2	54.9	30.6	54.0
H	√	√	√	**34.3**	**57.1**	**33.8**	**56.6**

**Table 6:** Detection performance with or without the CBCD module on different categories.

**Categories**	**Methods**	**Private Data**			**ComparisonDetector Data**		
		**QR**	**AP**	**AR**	**QP**	**AP**	**AR**
SCC	wo/CBCD	/	/	/	5.3%	18.2	33.9
	w/CBCD		/	/		18.9	36.8
HSIL	wo/CBCD	11.7%	16.4	32.9	75.5%	31.1	58.6
	w/CBCD		21.3	34.7		31.6	57.3 **↓**
ASC-H	wo/CBCD	11.0%	13.1	28.7	10.2%	16.8	31.2
	w/CBCD		18.2	29.3		17.6	34.5
LSIL	wo/CBCD	17.7%	44.5	69.4	4.1%	34.7	64.5
	w/CBCD		44.8	69.8		35.7	68.2
ASC-US	wo/CBCD	59.6%	31.2	60.2	4.9%	26.9	51.5
	w/CBCD		33.2	58.6 **↓**		27.4	52.9

**Table 7:** Comparative experimental results with existing cell detection networks.

Networks	Private Data		ComparisonDetector Data	
	AP	AR	AP	AR
Faster R-CNN [40]	29.8	55.2	28.9	54.4
YOLOV3 [43]	28.6	54.3	27.7	53.9
Comparison Detector [35]	32.2	55.6	30.8	54.3
SCAC Detector [33]	33.7	56.4	33.1	55.5
TDCC-Net [34]	33.5	56.9	32.2	55.2
PGCC-Net (ours)	**34.3**	**57.1**	**33.8**	**56.6**

**Table 8:** *p*-values of McNemar’s test.

Networks	*p*-Value	
	Private Dataset	ComparisonDetector Dataset
Faster R-CNN [40]	0.0125	0.0237
YOLOV3 [43]	<0.001	0.0092
Comparison Detector [35]	0.0021	0.0116
SCAC Detector [33]	<0.001	<0.001
TDCC-Net [34]	0.0362	0.0143

**Table 9:** Detection performance with different augmentation methods on the ComparisonDetector dataset.

Network	Augmentation Methods	AP	AP_50_	AR
Faster R-CNN	/	18.8	36.9	40.3
Faster R-CNN	Color	18.1	35.5	42.2
Faster R-CNN	Shape	27.2	51.6	51.4
Faster R-CNN	Hybrid	25.8	51.7	**56.6**
Faster R-CNN	Shape + Hybrid	29.7	55.5	56.4
Faster R-CNN	Color + Shape + Hybrid	**30.6**	**57.3**	54.4

**Table 10:** The AP of cell detection by different transformations on the ComparisonDetector dataset.

Network	Augmentation Methods	SCC	HSIL	ASC-H	LSIL	ASC-US
Faster R-CNN	/	7.0	21.2	8.4	21.7	17.7
Faster R-CNN	Color	7.0	18.4	7.1	19.8	12.5
Faster R-CNN	Shape	15.7	24.4	13.8	32.3	24.3
Faster R-CNN	Hybrid	**18.1**	25.0	12.1	31.6	27.2
Faster R-CNN	Shape + Hybrid	13.9	**29.4**	15.7	**33.9**	31.6
Faster R-CNN	Color + Shape + Hybrid	16.0	29.3	**16.8**	33.2	**31.7**

**Table 11:** The AR of cell detection by different transformations on the ComparisonDetector dataset.

Network	Augmentation methods	SCC	HSIL	ASC-H	LSIL	ASC-US
Faster R-CNN	/	18.4	60.6	19.6	58.4	51.6
Faster R-CNN	Color	20.2	**64.4**	22.3	56.6	42.7
Faster R-CNN	Shape	31.3	48.5	28.7	50.6	39.4
Faster R-CNN	Hybrid	**52.0**	59.0	**29.7**	55.8	53.2
Faster R-CNN	Shape + Hybrid	25.4	59.3	25.0	**60.2**	**56.3**
Faster R-CNN	Color + Shape + Hybrid	31.6	55.3	25.2	58.4	54.4

**Table 12:** Detection performance of different classes on the ComparisonDetector dataset.

Groups	Classes	Network	AP	AR
I	5-class (TBS standard)	Faster R-CNN	28.9	54.4
J	2-class (epidermal cells, basal cells)	Faster R-CNN	30.0	55.7
K	2-class (individual cells, cell clusters)	Faster R-CNN	32.9	56.1

**Table 13:** TP, TP, and FN for different detection networks.

Networks	Private Data			ComparisonDetector Data		
	TP ↑	FP ↓	FN ↓	TP ↑	FP ↓	FN ↓
Faster R-CNN [40]	16,532	4312	3139	6349	493	2308
YOLOV3 [43]	14,866	5367	4805	7256	635	1401
Comparison Detector [35]	17,627	3577	2044	7718	468	939
SCAC Detector [33]	18,133	3209	1538	8033	409	624
TDCC-Net [34]	17,963	2934	1708	8091	413	566
PGCC-Net (ours)	18,526	2561	1145	8236	405	421

## Data Availability

The data presented in this study are available on request from the corresponding author.
